# 

**Published:** 1859-01-01

**Authors:** 


					THE JOURNAL
Of
PSYCHOLOGICAL MEDICINE
MENTAL PATHOLOGY.
EDITED BY
FORBES WIN SLOW, M.D., D. C. L. Oxon.
o
? YOL. XII.
c; i
LONDON:
JOHN CHURCHILL, NEW BURLINGTON STREET.
MDCCCLIX.
/
Oo
w
3o 851 N
LONDON :
SAVILL AND EDWARDS, PRINTERS, CHANDOS STREET,
COVENT GARDEN.

				

